# The Application of Submerged Modules for Membrane Distillation

**DOI:** 10.3390/membranes10020025

**Published:** 2020-02-06

**Authors:** Marek Gryta

**Affiliations:** Faculty of Chemical Technology and Engineering, West Pomeranian University of Technology in Szczecin, ul. Pułaskiego 10, 70-322 Szczecin, Poland; marek.gryta@zut.edu.pl

**Keywords:** membrane distillation, submerged module, capillary membrane

## Abstract

This paper deals with the efficiency of capillary modules without an external housing, which were used as submerged modules in the membrane distillation process. The commercial hydrophobic capillary membranes fabricated for the microfiltration process were applied. Several constructional variants of submerged modules were discussed. The influence of membrane arrangement, packing density, capillary diameter and length on the module performance was determined. The effect of process conditions, i.e., velocity and temperature of the streams, on the permeate flux was also evaluated. The submerged modules were located in the feed tank or in the distillate tank. It was found that much higher values of the permeate flux were obtained when the membranes were immersed in the feed with the distillate flowing inside the capillary membranes. The efficiency of submerged modules was additionally compared with the conventional membrane distillation (MD) capillary modules and a similar performance of both constructions was achieved.

## 1. Introduction

The membrane processes are carried out in the membrane modules, usually composed of the membranes assembled in a tubular housing. Such a configuration enables the desired cross-flow velocity under different conditions of pressure and flow rate in order to control the concentration polarization and to achieve the appropriate values of the transmembrane pressure (TMP). In Japan (1988–1989) an idea was presented in which the bundles of submerged hollow fibres were assembled inside a non-pressure vessel and the filtration was realized by the removal of permeate by suction [1]. This low-pressure variant of micro- and ultrafiltration (MF and UF) was commercialised and applied in the water and wastewater industry [1,2,3].

The submerged modules concept is well established for the filtration of solutions containing a substantial quantity of suspended solids. The low values of TMP (usually below 0.05 MPa) limit fouling through the achievement of operating conditions close to the critical flux [4,5]. With regard to this, the submerged modules are employed in the membrane bioreactors (MBR) [6,7,8]. In this case, the membranes are usually assembled in the cassette system with feeding of air from the bottom of the cassette [1,8]. Air bubbling induces the fibre movement and the flow of feed along the membranes, which generates the surface shear, mitigating the deposition of a fouling layer [1,7]. Moreover, a periodic backwashing and additional chemical cleaning is employed for the removal of deposits from the membrane surface, thus enabling its long-term exploitation [4,7,8,9,10]. 

In membrane distillation (MD) the water evaporates through the pores of nonwetted, hydrophobic membranes. In this process, the driving force for mass transfer is a vapour pressure difference, but not the TMP. Therefore, the reasons for application of the submerged modules in the MD process are different than those in the case of MF or UF processes. The permeate collected during the single flow of the feed through the MD module constitutes only a few percent of feed volume filling the module channels. With regard to this, in order to achieve a higher degree of water recovery, the feed is circulated in the closed loop and simultaneously reheated before the feed water enters the module [11,12]. These operations are omitted by immersing the capillary membranes inside the feed tank. Thus, the application of submerged modules significantly simplifies a construction of installation, which also additionally reduces the heat losses in the MD process [13,14,15].

During a long-term exploitation of the MD module, the water fills a fraction of the membrane pores, thereby the elimination of membrane wettability becomes a very important issue [16,17,18]. The MD process is not pressure-driven, however, the hydrostatic pressure is necessary in order to obtain the feed flow through the module channels. Although the value of this pressure is usually not high, this is one of the reasons accelerating the membrane wetting, especially if the surface tension of the feed solution is low [17,18]. The immersion of membranes inside a non-pressure feed vessel allows elimination of a hydrostatic pressure generated by feed pumping.

Fouling is also one of the reasons causing the membrane wetting [19,20,21]. The application of air bubbling is commonly employed in the MBR in order to mitigate the fouling phenomenon and it also proves to be effective in the MD process [15,22,23]. Moreover, in the case of the presence of surface active agents, they are accumulated on the surface of air bubbles, which additionally limits the influence of these substances on the pore wetting [23]. Such a concept was successfully used in the MD process applied to the treatment of wastewater from the petrochemical industry and to the treatment of produced water generated during the extraction of gas and petroleum [2,24]. 

The driving force in the MD process does not directly depend on the bulk feed temperature, but on the feed/membrane interfacial temperature [25,26,27,28,29,30,31]. Therefore, the hydrodynamic conditions are important since the occurrence of temperature polarization reduces in a significant degree the MD process efficiency [30]. Bubble aeration and mixing allow the cross-flow of feed through the membrane bundle [32], however, it is difficult to obtain such a high flow turbulence in the submerged modules as in the case of the conventional cross-flow module with housing [30,33,34]. Using a Vacuum Membrane Distillation (VMD) variant can enhance the values of the driving force in this case [26], however, the application of pressure difference (vacuum) also accelerates the membrane wetting [13,30]. On that account the submerged modules working in a Direct Contact MD (DCMD) variant were utilized in the presented work. 

Due to the heat and mass transfer, the feed temperature in the boundary layer decreases rapidly, which results in a significant decline of the permeate flux. On that account, the turbulent/mixing conditions are important, especially when a higher feed temperature is used [35]. The submerged modules in the capillary configuration possess only one flow channel in the lumen side. The distillate usually flows through this channel; therefore, a movement (e.g., stirring) of the liquid filling the feed tank can decrease the negative effect of temperature polarization on the feed side. In this case, the feed flow in the submerged module system can be induced by the air bubbles introduced between the fibres. The effect of liquid movement in the feed tank can be also obtained by mechanical mixing [32] or by pumping of feed (recirculation) [36].

An enhancement of flow turbulence on the feed side limits the negative effect of the temperature polarization [34,35,36,37,38,39,40]. However, the bulk temperature is also decreased during the MD process, hence, the achievement of higher degrees of water recovery will require the energy supply also in the case of application of the submerged MD modules. The location of elements for heat transfer directly under the modules in the feed tank is advantageous. Such a concept limits the heat losses and enhances the feed flow turbulence between the membranes through the formation of the convection currents. In this case, a high effectiveness of heat transfer for the cross-flow system can be achieved by assembling the membranes in a horizontal position. Other concepts include the feed circulation in a loop from the feed tank to the external heat exchangers. In such a case, a significant influence on the process effectiveness includes a kind of energy source and a connection method of the MD installation with heat exchangers [41].

The advantages of MD submerged modules render them suitable for the application in MBR or in the MD process integrated with crystallization (MDC) and forward osmosis [27]. The studies of MD submerged modules presented in the literature were conducted using the capillary membranes of short length, usually not exceeding 20–30 cm. Therefore, a further development of the MD process requires additional testing of these modules also on a larger scale [26,34]. The submerged modules fabricated from capillary polypropylene membranes are currently offered by the ZENA Membranes company from the Czech Republic [42]. Besides the smaller laboratory modules, the company also produces larger submerged modules with a working membrane area of over 4 m^2^, which can be utilized for building the MD pilot installations. The size of the modules, especially the length of the capillaries and their packing density, has a decisive impact on the MD process. For this reason, the effect of module construction, capillary membrane arrangement and location in the feed tank, and the MD process conditions on the module performance was examined in the presented work.

## 2. Heat and Mass Transfer 

**Figure 1:**
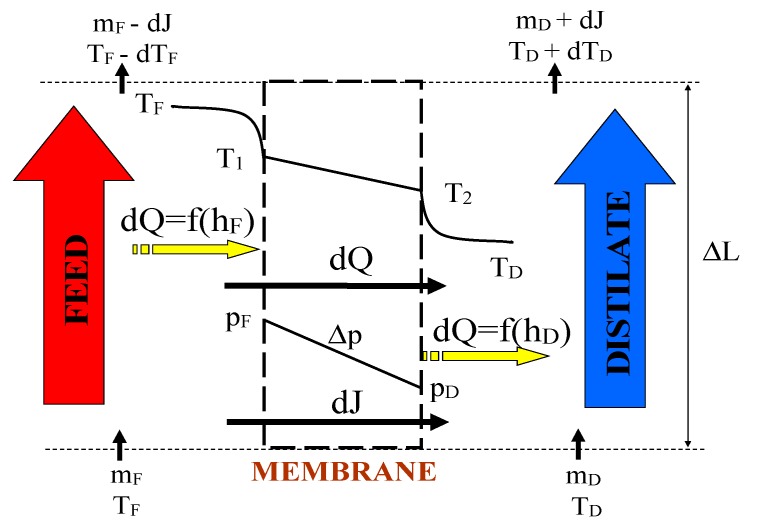
Scheme of heat and mass transfer in membrane distillation (MD) process. m_i_—mass flow rate [kg/s].

In the DCMD variant, a hydrophobic membrane separates the hot feed from the cooled distillate (Figure 1). The water vapour diffuses through the gas phase, filling the membrane pores, and a difference of vapour partial pressure (Δp) creates the driving force for mass transfer. The value of Δp depends on the temperature of the evaporation surface (T_1_) and condensation surface (T_2_).

The vapour pressure p_i_ of water for the diluted solutions can be determined from the Antoine equation [43]:

(1) $${\log\;p}_{i}\;=\;8.07131-\frac{1730.63}{233{.42\;+\;t}_{i}}$$

where p_i_ [mmHg] and t_i_—temperature (°C).

The effect of membrane morphology and the operating conditions of the MD process on the magnitude of the obtained permeate flux (J) is often described by equation [44]:

(2) $$J=\frac{\varepsilon }{\chi s}\frac{M}{{\mathrm{RT}}_{m}}D_{\mathrm{WA}}P\;\ln\frac{P-p_{D}}{P-p_{F}}$$

where p_F_ and p_D_ are the partial pressures of the saturated water vapour at the interfacial temperatures T_1_ and T_2_, on the feed and distillate side, respectively; ε—porosity, χ—tortuosity (in this work was assumed χ = 2), and s—membrane thickness. Moreover, P is process pressure, M—molecular mass, R—gas constant, T_m_—average membrane temperature, and D_WA_—the effective diffusion coefficient of water vapour through the membrane pores. 

The value D_WA_ was estimated based on the molecular (D_M_) and Knudsen (D_K_) diffusion coefficients:

(3) $${\frac{1}{D}}_{\mathrm{WA}}\;=\;{\frac{1}{D}}_{M}\;+{\frac{1}{D}}_{K}\;$$

where:

(4) $$D_{K}\;=\;\frac{d_{P}}{3_{}}\sqrt{\frac{{8\mathrm{RT}}_{m}}{\pi_{}M}}$$

(5) $$D_{M}\;=D\;{\left(\frac{T_{m}}{273.15}\right)}^{1.75}$$

and water vapour diffusion in air (D) is equal to 0.297 × 10^−4^ m^2^/s [45].

The interfacial temperatures T_1_ and T_2_ are different from the measured bulk temperatures on the distillate and feed side. This phenomenon is called the temperature polarization, the occurrence of which reduces the value of the driving force for mass transfer in the MD process [39,40]. The temperatures T_1_ and T_2_ were calculated using numerical modelling of the MD process [26,27]. In the presented work, a model described in the previous papers [44,46] was used for MD process simulations. In this model, the interfacial temperatures were calculated using the following equations:

(6) $$T_{1}=\frac{k_{m}{(T}_{D}+\frac{h_{F}}{h_{D}}T_{F})+h_{F}T_{F}-J\Delta H}{k_{m}+h_{F}(1+\frac{k_{m}}{h_{D}})}$$

(7) $$T_{2}=\frac{k_{m}{(T}_{F}+\frac{h_{D}}{h_{F}}T_{D})+h_{D}T_{D}+J\Delta H}{k_{m}+h_{D}(1+\frac{k_{m}}{h_{F}})}$$

where k_m_ is the heat transfer coefficient through the membrane [W/mK], h is the convective heat transfer coefficient [W/m^2^K], and ΔH is vapour enthalpy [kJ/kg]. The coefficient h was calculated from equation:

(8) $$h_{}\;=\;\frac{{\mathrm{Nu}\;k}_{L}}{d_{h}}$$

where Nu is the Nusselt number, k_L_—liquid (feed or distillate) heat transfer coefficient, and d_h_—hydraulic diameter of module channel. The value of Nu number was calculated from correlation [46]:

(9) $$\mathrm{Nu}=4.36+\frac{0.036\mathrm{Pe}\frac{d_{h}}{L}}{1+0.0011{\left(\mathrm{Pe}\frac{d_{h}}{L}\right)}^{0.8}}$$

where Pe is the Peclet number, L—length of channel (e.g., membrane capillary).

The above equations show that the temperatures T_1_ and T_2_ significantly depend on the flow turbulence; hence, in addition to the T_F_ and T_D_ values the MD process performance is also strongly affected by the flow velocity of the streams and the dimensions of the channels in a module.

**Figure 2:**
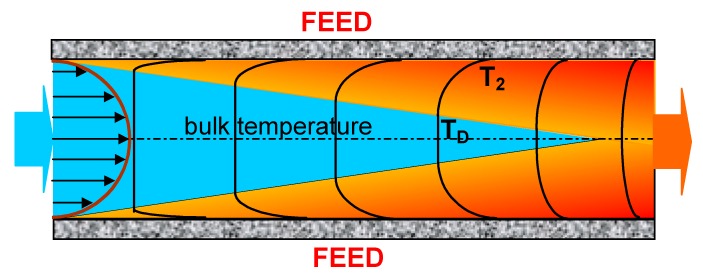
Scheme of distillate temperature changes during distillate flow inside the capillary membrane. T_D_—distillate temperature.

A temperature difference (T_F_ − T_D_) causes the distillate temperature to increase not only as a result of vapour condensation, but also due to the heat conduction through the membrane from feed to distillate stream. Therefore, a temperature of the boundary layer (T_2_) rises quickly, and the temperature in the distillate channel (T_D_) becomes close to the feed temperature (Figure 2). For this reason, it is important to determine the useful length of the MD module, which will depend not only on the flow velocity of the streams and their initial temperature [29,34,47], but also on the diameter of the capillary membranes.

The changes of the distillate and feed temperatures along the capillary membranes were calculated based on the balance equations formulated for a differential element (ΔL) presented in Figure 1:

(10) $$\left(v_{F[i]}A_{F}\rho_{F}-J_{\left[i\right]}\cdot \mathrm{dF}\right){\;\mathrm{Cp}}_{F}{}_{}\left(T_{F[i]}-T_{F[i+1]}\right)=h_{F}\mathrm{dF}\left(T_{F[i]}-T_{1[i]}\right)$$

(11) $$\left(v_{D[i]}A_{D}\rho_{D}+J_{\left[i\right]}\cdot \mathrm{dF}\right){\;\mathrm{Cp}}_{D}\left(T_{D[i+1]}-T_{D[i]}\right)=h_{D}\mathrm{dF}\left(T_{2[i]}-T_{D[i]}\right)$$

where ρ is the liquid density, dF is the membrane area for capillary length of ΔL, and A is the cross-section area of channel [m^2^] for the feed and distillate sides, respectively.

The presented MD model was experimentally validated in our previous works [44,46]. The numerical simulations were realized using distilled water as a feed, applying the physical-chemical parameters (such as C_P_, ρ, ΔH, and k_L_) published in work [48].

## 3. Materials and Methods 

**Figure 3:**
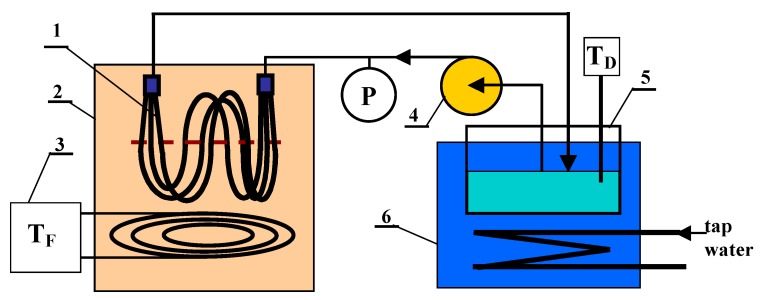
MD process experimental set-up. 1—submerged MD module, 2—feed tank, 3—feed temperature regulator with electric heater, 4—peristaltic pump, 5—distillate tank, 6—cooling bath, T—thermometer, P—pressure gauge.

The submerged modules were tested using the DCMD variant in the installation schematically shown in Figure 3. In most cases, the membrane module was located in the feed tank and the distillate flowed inside the capillary membranes. For comparison, the tests were also carried out in which the module was immersed in a distillate tank and the feed flowed inside the capillaries. The volume of feed and distillate tanks were 4 and 1 L, respectively. The maximum efficiency of the peristaltic pump (type 372A, Elpin-Plus, Lubawa, Poland) was 0.5 L/min, which allowed examination of the submerged modules with the flow rate in the range of 0.2–1.4 m/s (depending on module construction). The hydrostatic pressure induced by the water flow was measured at the module inlet using a pressure gauge (KMF, Wika, Włocławek, Poland) with an accuracy of ±100 Pa.

In the conducted MD studies, the distillate temperature was 293 ± 1 K and the feed temperature was varied in the range of 323–358 K. An electric heater with electronic temperature regulator (± 0.5 K) was applied (type EDIG, Nűga Company, Georgensgmünd, Germany). The electronic thermometers (PT 401, Elmetron, Poland) were used to measure the temperatures with an accuracy of ±0.1 K. Distilled water with the addition of NaCl (1 g/L) was used as the feed, which allowed checking of feed leakage. The value of electrical conductivity (6P Ultrameter, Myron L Company, Carlsbad, CA, USA) of water on the distillate side did not exceed 10 µS/cm in each of the examined cases, which proved that the module construction was tight.

Most MD tests were performed without mechanical mixing of the feed, and the feed flow between the capillaries was caused only by the natural convection generated by the electrical heater assembled below the submerged module (Figure 3). Moreover, to demonstrate the effect of temperature polarization on the external side of the capillaries, the additional experiments were carried out with mixing of water in the feed tank (No. 2 in Figure 3) by the magnetic stirrer (700 rpm, RCT type, IKA-Werke GmbH, Staufen, Germany).

**Figure 4:**
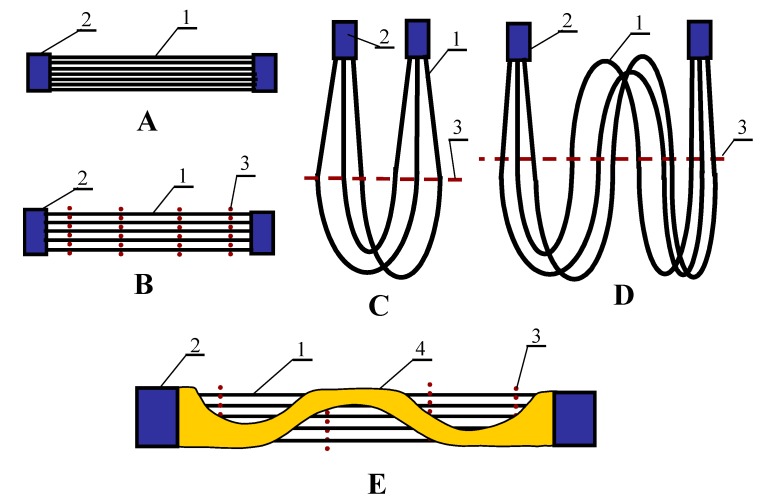
Various constructions of submerged MD module. (**A**) statistical arrangement of capillaries; (**B**) capillaries located inside the nets; (**C**) U-shape type, membranes located inside the net; (**D**) multiple U-shape; (**E**) variant 3B stiffened by tubular housing with cut out large holes. 1—capillary membrane, 2—module head, 3—PP grid, 4—tubular housing with holes.

The commercial capillary membranes fabricated for the microfiltration process (MF) were used to construct the submerged modules. The capillaries were made from different polymers, and membranes exhibited the significant differences in their parameters (Table 1). These membranes were assembled in modules of various constructions that are schematically shown in Figure 4. The parameters of tested modules are presented in Table 2.

To compare the performance of submerged modules with the conventional modules, additional MD tests were performed using the capillary modules (Table 3). The membranes inside the modules with housing were assembled in grids, i.e., similarly to the modules shown in Figure 4B,E. This mode provided good hydrodynamic conditions for liquid flow along the capillaries, which allowed significant increase of the efficiency of capillary modules [49].

## 4. Results and Discussion

### 4.1. Influence of MD Process Parameters 

In the case of submerged modules there are two options of MD process operation. In the first option (Mode 1), the modules are submerged in a feed tank and the distillate flows inside the capillaries. In Mode 2 the configuration is reversed, i.e., the modules are submerged in the distillate tank. In each of these modes the possibility of enhancement in the flow turbulence mainly exists in the lumen of the capillaries. In this work, the majority of studies were carried out using Mode 1, in which the modules were immersed in the hot feed. Moreover, the additional series of measurements was also performed with Mode 2 for comparison purposes.

**Figure 5:**
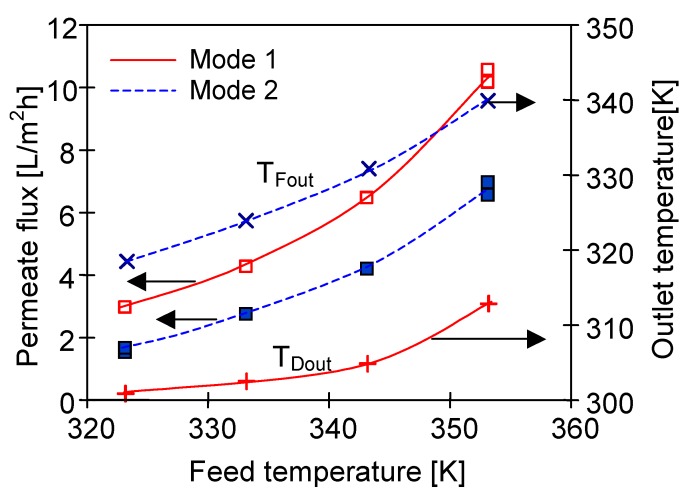
The influence of feed temperature and direction of feed flow (Mode 1 or Mode 2) on the permeate flux and outlet temperature of feed or distillate. Module SMD1. Flow rate inside the capillaries v = 0.62 m/s. Distillate inlet temperature 293 K.

An advantage of submerged MD modules is maintaining a constant temperature outside the membranes, but the temperature of stream flowing in the lumen undergoes the changes (feed cools down or distillate heats up), which decreases the process efficiency. With regard to an exponential dependence of vapour pressure on a temperature, it can be expected that the changes of feed temperature will have a larger influence on a decline of process efficiency. The results presented in Figure 5 confirmed that the permeate flux was almost two times higher when the submerged MD module was placed in the feed tank (Mode 1) compared to the distillate tank (Mode 2). In the former case the entire surface of the capillaries was in contact with the feed at the constant high temperature (e.g., 353 K). In the latter option (Mode 2), the feed flowed inside the capillaries and the feed temperature systematically decreased along the module length due to the mass and heat transfer. As a result, the outlet feed temperature (T_Fout_) was lower from its inlet value (T_Fin_) and a decline of the permeate flux was observed (Figure 5). 

**Figure 6:**
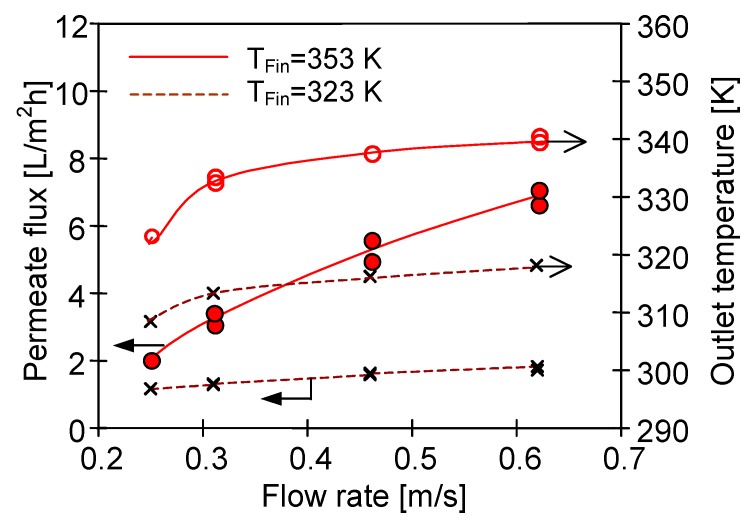
The influence of feed flow velocity on the permeate flux and feed outlet temperature for two different inlet temperatures (T_Fin_). Mode 2—distillate temperature 293 K, feed inlet temperature 323 K or 353 K. Module SMD1.

A decrease in the feed temperature (Mode 2) can be also limited by increasing the flow rate of the feed, which allows the permeate flux to increase, especially for a higher inlet feed temperature (Figure 6). However, the studies confirmed that even for high flow rates, it was impossible to prevent the temperature decline of the feed flowing inside the capillaries. In this case, a significant part of the advantages associated with the increase in the flow rate resulted from the increasing turbulence of flow, which limited the negative effect of temperature polarisation [29,40,47,50]. Since the hydrostatic pressure increased with the increase in the flow rate, the possibility of membrane wetting was also enhanced in this case.

The above-presented results confirmed that a placement of submerged modules in the feed tank was more advantageous. In this configuration (Mode 1) the distillate flowed inside the capillaries, and the distillate temperature gradually increased, moreover, such an option reduced the process efficiency to a smaller degree (Figure 5). This resulted from the character of changes in the driving force, which in the DCMD was the vapour pressure difference (Δp) between the feed and distillate [39,51,52]. The water vapour pressure increased exponentially, exhibiting a significant growth just only above 333 K [39,43,50]. Therefore, the increase in the distillate temperature, even from 303 to 333 K (ΔT = 30 K, Δp = 15.6 kPa), did not cause such a large decrease of the driving force as the decline of feed temperature from 353 to 323 K (ΔT = 30 K, Δp = 35 kPa). Therefore, the MD efficiency obtained for Mode 1 (e.g., T_F_ = 343 K) was similar to that obtained for higher feed temperature (T_Fin_ = 353 K, v_F_ = 0.62 m/s) with the feed flowing inside the capillaries (Figure 5, Mode 2). 

**Figure 7:**
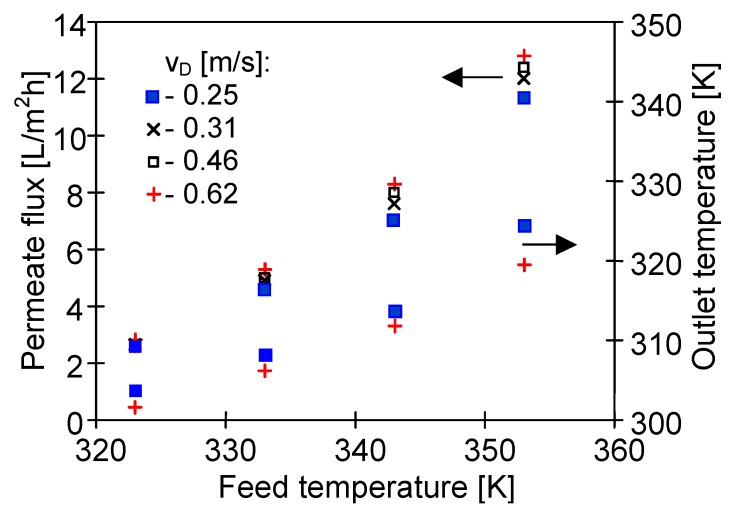
The influence of feed temperature and the distillate flow rate on the permeate flux and distillate outlet temperature. T_Din_ = 293 K. Mode 1. Module SMD2.

In an analogous manner, the increase of the distillate flow rate can limit an elevation of distillate temperature inside the capillaries. Due to the abovementioned dependence of the vapour pressure on temperature, a slight growth of the permeate flux (as a function of flow rate) was obtained, mainly for the distillate outlet temperature in the range of 313–327 K (Figure 7). On that account, it was advantageous to select such a flow rate of distillate that allowed a distillate outlet temperature below 313 K. In the case of the tested module SMD2 such flow rate was above 0.4 m/s (Figure 7).

**Figure 8:**
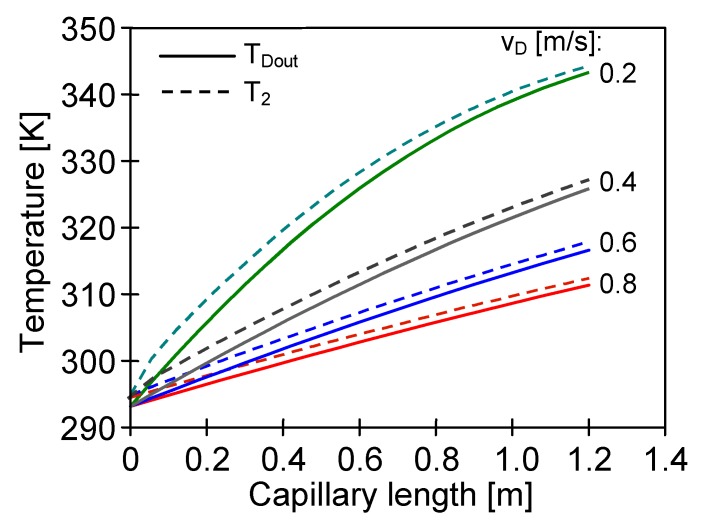
The results of numerical calculations. The influence of distillate flow rate on distillate bulk temperature and temperature of condensation layer (T_2_). Mode 1: T_F_ = 353 K, T_Din_ = 293 K. Membranes: Accurel PP S6/2, L = 120 cm. Feed flow v_F_ = 0.05 m/s.

**Figure 9:**
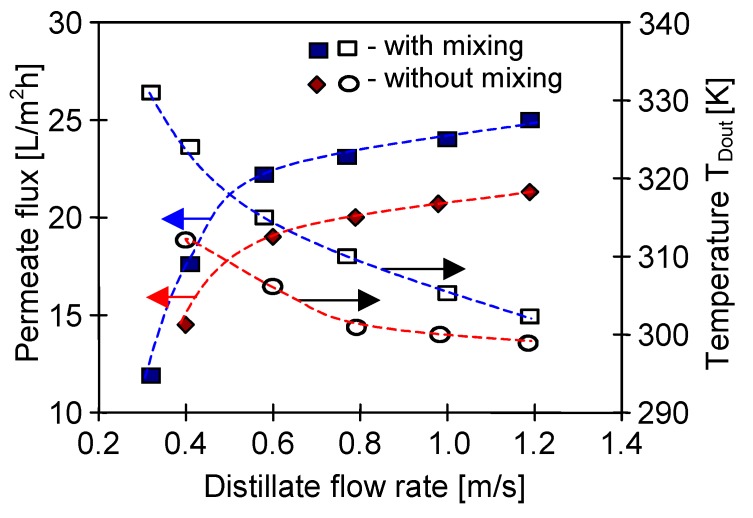
The influence of distillate flow rate and turbulence in the feed tank on the permeate flux and distillate outlet temperature in the presence and absence of feed mixing. Mode 1: T_F_ = 353 K. Module SMD12 (Accurel PP S6/2, L = 120 cm).

The numerical modelling revealed that the negative effect of the increase of the distillate temperature would be enhanced with the increasing length of capillary membranes. The simulation results for Accurel PP S6/2 membranes with a length of 120 cm are presented in Figure 8. An extension of the module length from 80 to 120 cm caused the temperature T_Dout_ to increase, e.g., for v_D_ = 0.6 m/s from 309.5 to 316.6 K. The calculation results also confirmed the significant effect of the flow rate on the value of temperature polarization. The difference between T_2_ and T_D_ was reduced more than twice by increasing the velocity v_D_ from 0.2 to 0.8 m/s. The results of experimental studies confirmed the conclusions resulting from the numerical simulations. The performance of the SMD12 module shown in Figure 9 (length 120 cm) proved a greater influence of distillate flow rate changes than that observed for a shorter SMD2 module (Figure 7).

In the MD process the feed is a source of energy, hence, for the continuous separation it is necessary to ensure a minimum feed flow along the fibres of the submerged modules. In the calculations presented in Figure 8 it was assumed that the feed flows at a velocity of 0.05 m/s along the membranes (e.g., due to the air bubbling or feed recirculation) and that a distance between the capillaries is 7 mm. A significant influence of turbulence of the feed flow on the MD performance was confirmed by the results presented in Figure 9. Activation of a magnetic stirrer in the feed tank caused an increase of the SMD12 module efficiency by over 20%. Although the bulk temperature of feed along the entire surface of the membranes was similar, the temperature T_1_ was lower than T_F_, due to the water evaporation and heat conduction from the feed to the distillate (Figure 1).

### 4.2. The Effect of Membrane Morphology

**Figure 10:**
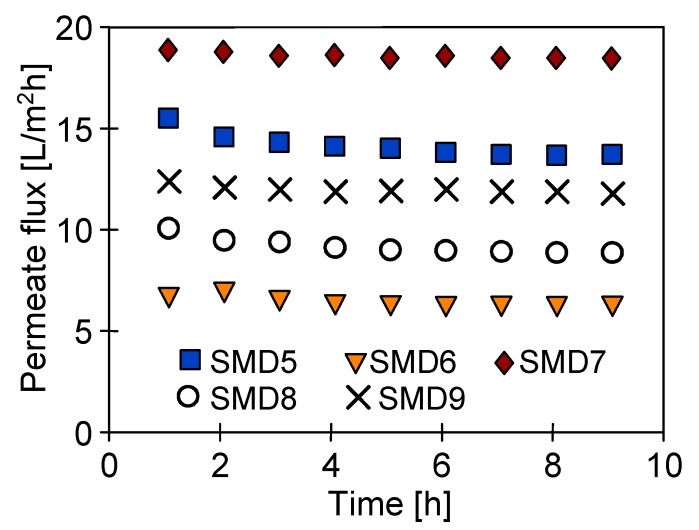
The influence of membrane type on the permeate flux. Mode 1: T_F_ = 353 K. Membranes: SMD5—Accurel PP S6/2; SMD6—Accurel PP V8/2 HF; SMD7—HL310; SMD8—PV370; and SMD9—K1800.

The membrane parameters, such as a pore size and wall thickness, will influence the quantity of energy transferred through the membrane [25,35]. Therefore, the temperature in the lumen of the capillary varies differently depending on the membrane type, which has an influence on the module yield as it was demonstrated. On that account, in this part of the study shorter modules (SMD5-9) were employed, allowing an outlet temperature closer to the inlet values. The obtained results of the studies are presented in Figure 10. In each tested case, the permeate flux was stable over the time of module operation. However, the flux values differed significantly for the particular modules. One of the main reasons was the different thickness of the membrane walls and various pore sizes. The highest efficiency (19 L/m^2^h) was achieved for the SMD7 module with the ECTFE membranes having the wall thickness of 170 µm and 0.4 µm pore size, whereas the lowest permeate flux (6.7 L/m^2^h) was obtained for PP membranes with the wall thickness of 1500 µm and 0.2 µm pore size. The results obtained for the membranes with similar capillary diameters and wall thickness (Table 1, SMD5 and SMD9) indicated that a slightly larger value of the permeate flux may result from a higher porosity and larger pore size [53,54]. For similar reasons the efficiency of the SMD7 module was higher than that obtained for the SMD8 module with PV370 membranes, which had the smallest pore diameters (0.1 µm) (Table 1).

The influence of membrane morphology and the operating conditions of the MD process on the efficiency of the modules presented in this work have been also reported by different authors in the studies of submerged MD configuration [13,15,24,30,31,32,33,55,56]. The results compiled in Table 4, especially a comparison of the permeate flux values, indicate that due to the limited turbulence in the feed flow, the efficiency of the submerged modules was at average level. For example, the permeate flux in the range of 4–13 L/m^2^h should be expected for submerged modules when the feed temperature is 343 K. Moreover, the experimental results presented above indicate that obtained performance can be increased if the proper hydrodynamic conditions are applied in the MD installation.

### 4.3. The Effect of Capillary Membrane Length

**Figure 11:**
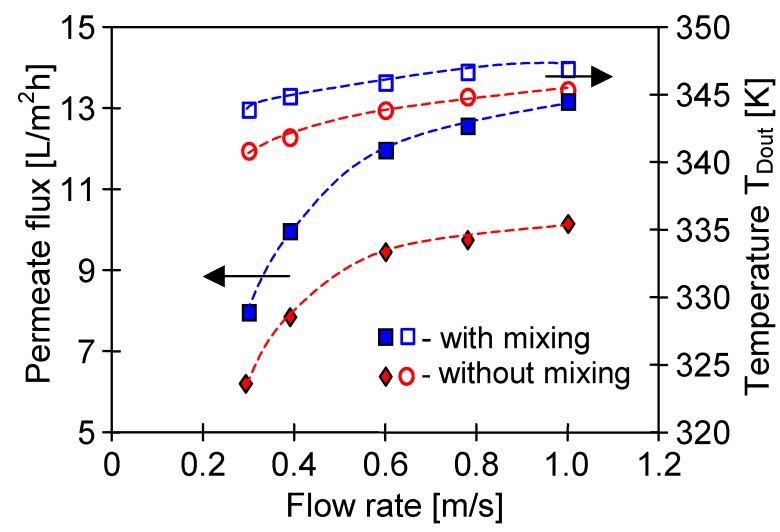
The influence of distillate flow rate and turbulence in the feed tank on the permeate flux and distillate outlet temperature in the presence and absence of feed mixing. Mode 1: T_F_ = 353 K. Module SMD4 (PV370, L = 120 cm).

Not only the structure of a membrane wall but also the diameter of a capillary has a significant influence on the performance of the MD submerged modules. In the case of membranes with a diameter of D_in_ = 1.8 mm, the distillate outlet temperature was much lower than the feed temperature after passing a 120 cm long capillary, even at lower distillate flow rates (Figure 7, Figure 8 and Figure 9). However, for capillaries with a smaller diameter, the temperature of distillate flowing inside the capillaries can approach the feed temperature (Figure 2), which will result in a large decrease in module efficiency. This dependence was confirmed by the results of the MD studies with SMD4 module (PV370 membrane, D_in_ = 0.37 mm) presented in Figure 11, for which the obtained permeate flux values were twice lower than those obtained for the SMD12 module with capillary membranes having the same length. Moreover, the permeate flux increased by over 25% when the magnetic stirrer in the feed tank was running, which indicated that the temperature polarization on the feed side had a large impact on the performance of the SMD4 module, similar to the case of the SMD12 module (Figure 9). A significant influence of feed agitation on the submerged modules performance was also presented in work [26]. 

**Figure 12:**
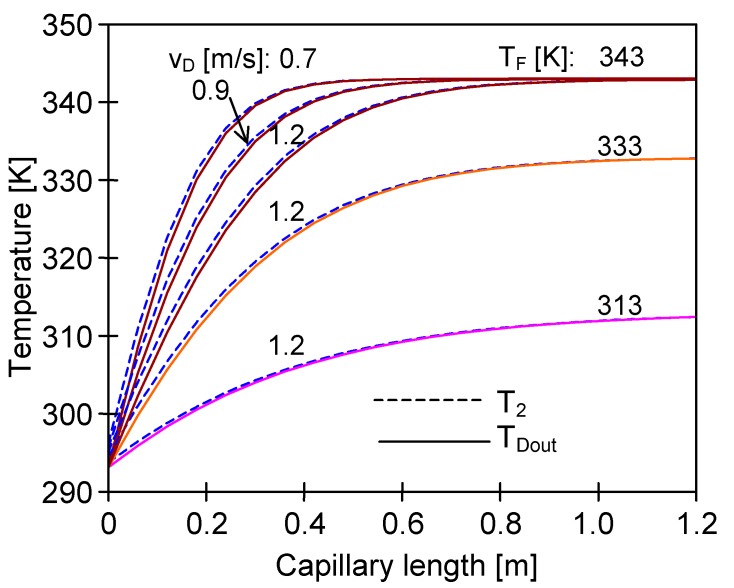
The influence of distillate flow rate and T_F_ on the distribution of the T_2_ and distillate outlet temperature along the membrane length. Mode 1. Membranes PV370, L = 120 cm.

**Figure 13:**
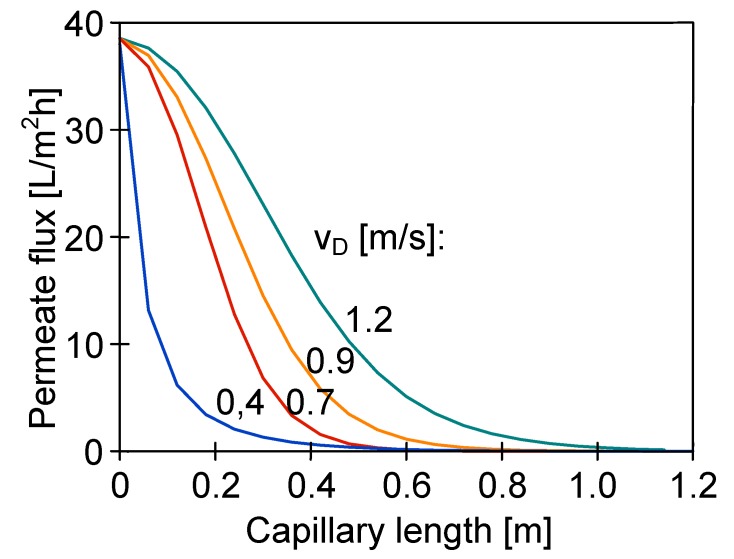
The influence of distillate flow rate on the distribution of the permeate flux along the module length. Mode 1. T_F_ = 343 K. Membranes PV370.

The numerical analysis of the MD process with PV370 membranes revealed that the distillate temperature inside capillaries was close to the feed temperature after flowing through a distance of 20–30 cm, even for high flow velocities (e.g., 1.2 m/s) (Figure 12, T_F_ = 353 K). Due to such a significant increase in the distillate temperature, more than half of the module working area was practically inactive (Figure 13). A large part of the membrane area was also inactive when the amount of energy transferred to the distillate was substantially reduced by decreasing the feed temperature to 313 K (Figure 12). Moreover, the distillate temperature was lower than 330 K in each of the analysed cases (T_F_ = 353 K) for capillaries having up to 20 cm length, which allowed the generation of the appropriate driving force for vapour transfer. For this reason, a good MD process performance can be obtained by using short (e.g., 10–20 cm) modules in laboratory tests. However, these results cannot be reproduced in the industrial installations with longer modules. A similar conclusion was also drawn by other authors [26].

Although an increase of the flow rate is an effective way of lowering the distillate temperature, this option simultaneously causes an increase of the hydrostatic pressure. In the case of PV370 membranes, for v_D_ = 1.2 m/s this pressure was increased to 0.14–0.16 MPa (module SMD4), which created a risk of exceeding of the Liquid Entry Pressure (LEP) value. Nevertheless, even at 0.18 MPa no leakage was observed. These results indicated that the obtained hydrostatic pressure was lower than LEP. However, it is worth mentioning that each increase of pressure accelerated a phenomenon of membrane wetting during the long-term module exploitation. Therefore, also for this reason, the capillary membranes with the diameters definitely larger than the tested PVDF membranes (D_in_ = 0.37 mm) should be applied in the submerged modules.

**Figure 14:**
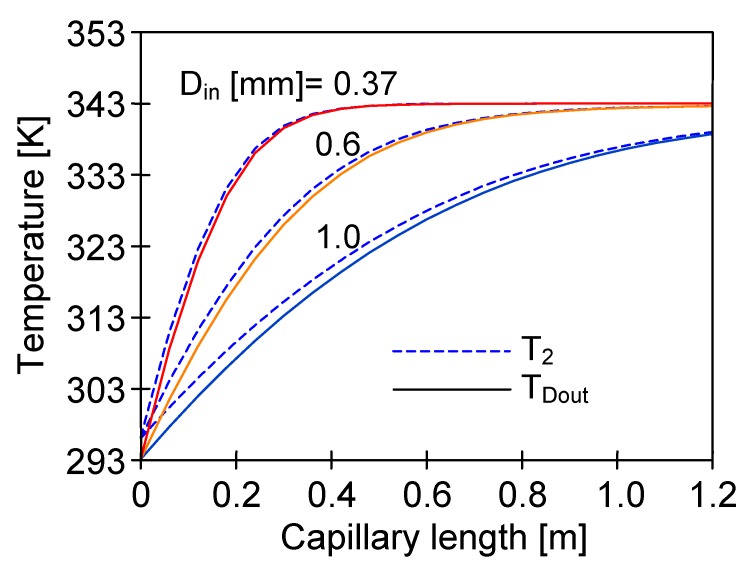
The influence of capillary diameter on the feed outlet temperature. Mode 1. Membrane PV370, T_Din =_ 293 K, T_F_ = 343 K, v_D_ = 0.7 m/s and v_F_ = 0.05 m/s.

The presented results indicate that determination of the appropriate diameter of the capillary membranes that can be used in the submerged MD modules depends on the process temperature and a required length of the module. Figure 14 shows the calculation results obtained for membranes with a diameter in the range of 0.37–1.0 mm. For the analyzed MD process conditions and the capillary membrane diameter equal to 1.0 mm, the obtained T_Dout_ value was about 10 K lower than T_F_ with a membrane length of 0.8–1 m. Therefore, the minimum diameter should be 1 mm for capillary membranes assembled in the industrial modules.

**Figure 15:**
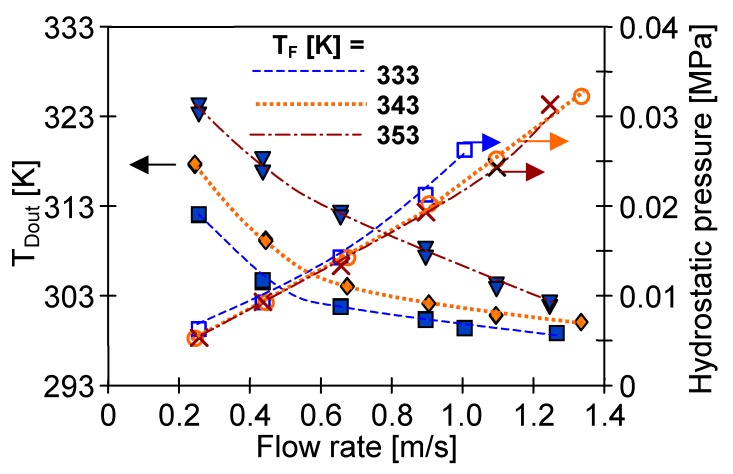
The influence of distillate flow rate on distillate outlet temperature and hydrostatic pressure inside capillaries. Module SMD3 (Membrane Accurel PP S6/2). Mode 1: T_F_ = 353 K.

The latter conclusion was confirmed by tests using the SMD3 module, in which 1.8-mm diameter membranes were used. In this case, the hydrostatic pressure increased only to 0.032 MPa, although the flow rate was increased to almost 1.4 m/s (Figure 15). Moreover, the application of the membranes with a larger diameter (over 1 mm) also allowed lower distillate temperature with the increase of flow rate. In the discussed experiments the T_Dout_ value approached the inlet value for a flow rate of over 0.6 m/s.

### 4.4. Module Construction

The results presented in Figure 5, Figure 6 and Figure 7 were obtained using U-shape modules with the membranes assembled in the spacing grids (Figure 4D). Such a solution achieved a distance of 3–10 mm between the membranes. Such significant distances permitted a free flow of the feed between the capillaries, hence, the effect of stream distributions on the process efficiency was eliminated. However, these modules had a small packing density, hence, a construction in which the membranes were assembled in the fibre bundles (e.g., Figure 4A), allowing definitely larger membrane areas calculated per unit volume of the feed tank. A basic issue will be the prevention from excessive decline of the feed temperature inside such a bundle due to a high resistance of the feed flow between the capillaries. Therefore, one of the important construction parameters is the distance in which the fibres should be placed from each other. The results obtained for the modules SMD7 and SMD8 are presented in Figure 10. In the SMD8 module the membranes were arranged at the distance of 0–1 mm, whereas in the SMD7 module the spacing grids were applied, and as a result, the distance between the capillaries amounted to 2–3 mm. The resulting lower permeate flux for the SMD8 module confirmed that a too-high packing density (PD) of fibres decreases the efficiency of the submerged MD module. A value of PD is usually expressed by a ratio of the capillary volume to the tank volume [26], which can be calculated from the following equation:

(12) $$\mathrm{PD}\;=\;\frac{{N\;D}_{\mathrm{out}}^{2}}{D_{V}^{2}}$$

where N—a number of capillaries, D_out_—external diameter of capillary membrane, D_V_—vessel diameter. The equation is valid when the capillaries are uniformly distributed in the tank.

**Figure 16:**
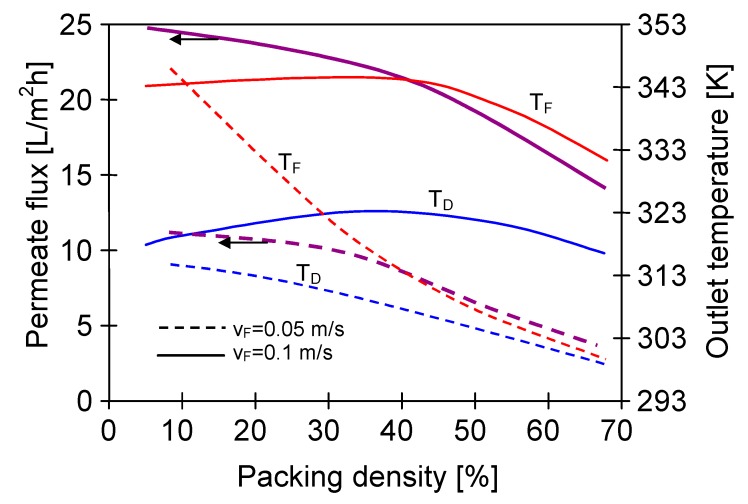
The influence of membrane packing density on the permeate flux and feed outlet temperature for various feed flow values. Mode 1. Distillate inlet temperature 293 K, feed temperature 353 K. v_D_ = 0.4 m/s. Membrane Accurel PP S6/2.

The results obtained from the numerical analysis, which takes into account the effect of membrane packing density on the submerged module performance, are shown in Figure 16. In these calculations it was assumed that the capillary membranes were vertically submerged in a cylindrical vessel with exemplary diameter equal to 5 cm. Moreover, the calculations were performed with the assumption that the feed flowed from the bottom of the tank along the membrane bundle (120 cm) at a velocity of 0.05 or 0.1 m/s, and with an initial temperature T_Fin_ = 353 K. In both cases, the flow rate of distillate was v_D_ = 0.4 m/s. The number of capillaries in the tank was increased from 20 to 250, which resulted in a change in packing density values from 5% to 68%. 

An increase of the packing density caused a deterioration of the module operating conditions, although the MD process efficiency increased more than twice for v_F_ = 0.1 m/s. It was found that even such a small increase in the flow rate hindered the reduction of the feed temperature T_F,_ as well as improved the conditions of water evaporation (increase of T_1_). The obtained results confirmed the conclusions of the previous experimental studies, which indicated that the membrane packing density at a level of 40% was advantageous for MD modules [49]. In the case of arrangement of the capillary membranes in the orthogonal system, the considered Accurel PP S6/2 membranes will be at a distance of about 2 mm from each other, if 40% packing density is applied.

### 4.5. Comparison of Submerged and Capillary MD Modules

**Figure 17:**
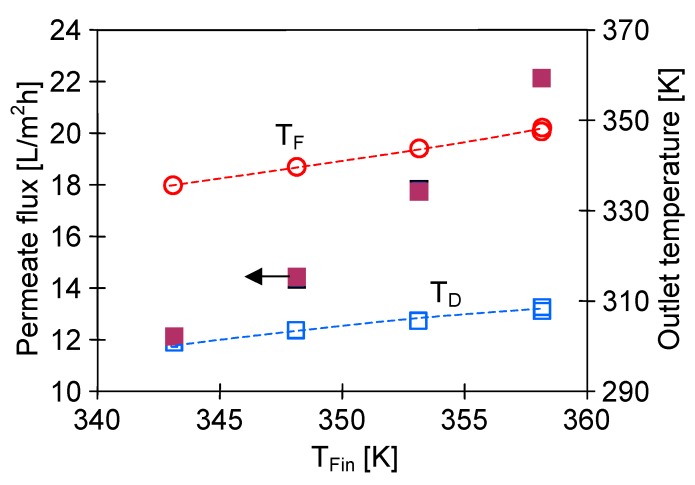
The influence of feed temperature on the permeate flux and feed/distillate outlet temperature. Capillary module CMD1. T_Din_ = 290 K, v_F_ = 0.8 m/s, v_D_ = 0.4 m/s.

**Figure 18:**
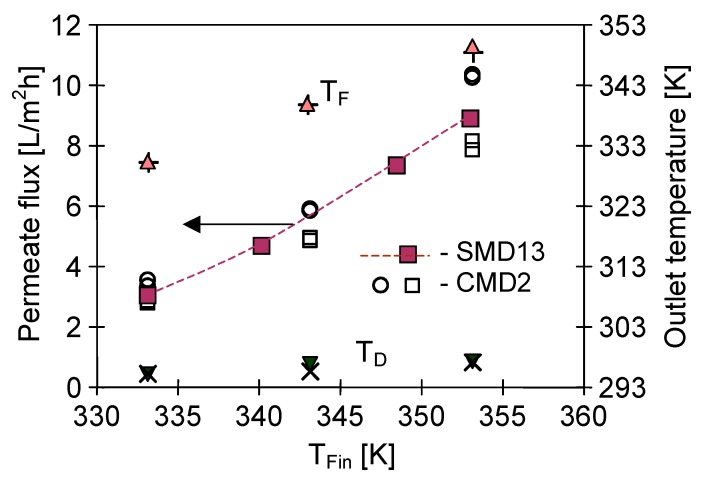
The influence of feed temperature on the permeate flux and feed/distillate outlet temperature. Capillary module CMD2. T_Din_ = 290 K, v_F_ = 0.6 m/s, v_D_ = 0.3 m/s. □▲▼—feed on the housing side; ○+x—feed inside the capillary. ■—flux obtained for SMD13 module (Mode 1).

The above results clearly indicate that both the diameter and length of the capillary membranes assembled in the MD submerged modules, as well as their packing density, were the important construction parameters. Maintaining the appropriate construction and operating conditions (feed turbulence and larger capillary diameter), it was possible to achieve an almost constant feed temperature outside the submerged modules. However, the temperature of distillate flowing inside the capillary membranes increased with the increase of module length. If the temperature of the distillate exceeded 320 K, this caused a significant decrease in the module efficiency (e.g., Figure 9, v_D_ = 0.3 and 0.4 m/s). In the conventional cross-flow MD module configuration with housing, the feed temperature decreased along the module; hence, the elevation of the distillate temperature in this module was smaller than that in the submerged modules (Figure 17). However, the temperature increase in the range of 315–320 K did not cause such a significant increase in the water vapour pressure. Therefore, when the distillate temperature was maintained at this level, the efficiency of the submerged module similar to that obtained in the conventional capillary modules can be achieved (Figure 9 and Figure 17). Heat conduction from the feed to the distillate (heat loss) can be reduced by using thick-walled membranes; however, in this case the mass transfer resistance was also increased, resulting in a decline in the process efficiency (Figure 18). The performance of the SMD13 submerged module in this case was similar to the CMD2 capillary module.

The efficiency of the MD process increased with the decrease of membrane wall thickness. Unfortunately, the mechanical strength of capillaries was reduced in this case. The membranes in the submerged modules were subjected to a significant stress, hence, reinforced membranes were used in the MF or UF industrial modules [1]. In the case of MD, one solution can be assembling the capillary membranes in supporting constructions, eliminating an excessive stress, particularly at a bonding layer in the module head. For example, a tubular housing as in the typical capillary module can be used. However, the housing will possess the holes, which enable a free flow of the feed from a tank to the space between the fibres (Figure 4E). In the examined case the SMD10 module was applied, in the housing of which three large holes were cut out, leading to a 40% loss of the housing area. The obtained MD permeate fluxes were compared with the results for the SMD11 module with the same membranes but without the external housing. A high and stable process efficiency was achieved in both cases (Figure 12). A slightly higher permeate flux obtained for the SMD10 module resulted most probably from the differences in the fibre arrangement in a module and from the fact that the fibres were shorter by about 15%. Moreover, such a result indicated that the application of the housing with the large holes did not deteriorate the MD process conditions. 

In the MD process a phase transition (water evaporation) takes place, hence, the process requires delivery of a large quantity of energy. For this reason, the thermal efficiency of the process is important, and it can be determined from the following relationship:

E = Q_D_/Q_T_ (13)

where Q_D_ is the latent heat of distillate and Q_T_ is the total heat transferred from feed to the distillate stream.

**Figure 19:**
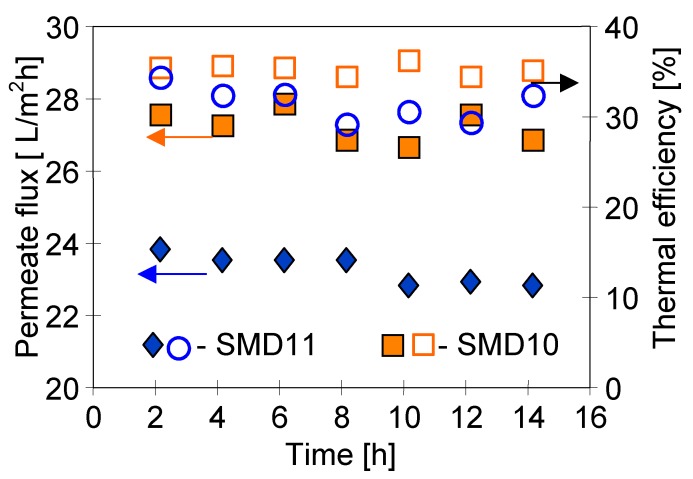
The influence of module construction on the permeate flux and thermal efficiency. Membrane PV370. Module design: SMD10—Figure 4E, SMD11—Figure 4B. Mode 1. T_F_ = 353 K, v = 0.75 m/s.

The calculated values of the thermal efficiency for submerged modules did not exceed 40% (Figure 19), and these values were significantly lower than those obtained for MD capillary modules with housing, which achieved the efficiency at a level of 70–80% [58]. In the performed studies (Figure 19), mixing of feed was not used, and the convective currents caused by heaters located under the modules only forced the feed flow. For this reason, it can be expected that a better efficiency of submerged modules is possible under enhanced flow turbulence on the feed side. One of the solutions is air bubbling applied in the industrial submerged MF or UF modules [1,30,38]. Aeration is also advantageous in the MD process [22,23], but one should remember that in certain cases the air bubbles can also initialize crystallization, which contributes to the membrane scaling [15,33]. Another option is the application of the mechanical mixing. In the case of the SMD12 module (Figure 9, v_D_ = 1–1.2 m/s), running the magnetic stirrer increased the thermal efficiency from 40% to 65%.

## 5. Conclusions

In the recent years, the interest in membrane distillation has significantly increased and several hundred of articles on MD were published. Nevertheless, only a few of them raise the issue of submerged modules, which can be an interesting alternative to the classical capillary modules with the housing. The results of the research described in the present paper demonstrate that a high MD process efficiency similar to the capillary modules can be also achieved using the submerged modules.

A placement of MD submerged modules in the feed tank allowed a constant feed temperature along the entire membrane surface. Hence, in that case the efficiency of the MD process was twice as high as in the configuration in which the feed flowed inside the capillaries and the modules were submerged in a cold distillate. A significant increase in the module efficiency can be achieved for the feed flowing between the fibres at a velocity of at least 0.05–0.1 m/s.

The application of high packing density of the membranes in the module will hinder the flow of feed between the capillaries, and as a consequence it can lead to a local drop of the feed temperature. The obtained results indicate that the packing density at a level of 40% is advantageous for submerged modules. Under these conditions, for tested capillary membranes (external diameter 2.6 mm) the distance between particular capillaries should amount to about 2–3 mm. 

A high feed temperature along the entire module surface causes a significant increase of the distillate temperature. When T_D_ value reaches 320–330 K, a significant decrease of the process efficiency is observed. Using the membranes with a diameter of at least 1 mm, the T_D_ increase can be limited by increasing the flow rate of the distillate over 0.4 m/s. For membranes with a diameter below 1 mm, the T_D_ becomes close to the feed temperature after the distillate flows through a 20–30 cm distance along the capillary, even for the high flow rates (1.2–1.4 m/s). In this case, more than half of the module area (for L = 120 cm) is not working and the permeate flux is close to zero in this zone.

In the case of the membranes with a small diameter an increase of distillate flow rate causes a significant growth of hydrostatic pressure, which can also accelerate their wetting. For example, for the membrane with a diameter of 0.37 mm, an increase of the flow rate from 0.4 to 0.78 m/s caused an increase of hydrostatic pressure from 65 to 155 kPa. The application of the capillary membranes with larger diameters allows definitely smaller values of pressure, e.g., for the tested membrane Accurel PP S6/2 with the diameter of 1.8 mm, the hydrostatic pressure was 33 kPa when flow rate was equal to 1.34 m/s.

## Figures and Tables

**Table 1:** Commercial capillary membranes assembled in submerged MD modules. Membrane parameters—manufacturers’ data.

Manufacturer	Membrane	Polymer	d_p_ [µm]	Porosity [%]	D_in_ [mm]	Wall Thickness [mm]
EuroSep Poland	EuroSep PP	PP	0.20	70	1.8	0.4
PolyMem Poland	K1800	PP	0.20	74	1.8	0.35
Membrana Germany	Accurel PP S6/2	PP	0.22	73	1.8	0.4
Membrana Germany	Accurel PP V8/2 HF	PP	0.20	73	5.5	1.5
Memtek, Australia	HL310	ECTFE	0.40	75	0.31	0.17
Memtek, Australia	PV370	PVDF	0.10	74	0.37	0.12

d_p_—pore diameter, D_in_—capillary membrane inner diameter.

**Table 2:** Parameters of submerged MD modules.

Module	Membrane	Number of Capillaries	Length [cm]	Area (Inside) [cm^2^]	Mode (Figure 4)
SMD1	EuroSep PP	3	85.5	145.0	D
SMD2	K1800	3	84.7	143.7	D
SMD3	Accurel PP S6/2	2	60.5	68.4	D
SMD4	PV370	19	120	265.3	D
SMD5	Accurel PP S6/2	4	22.5	50.9	C
SMD6	Accurel PP V8/2 HF	1	26.5	45.8	B
SMD7	HL310	75	13.0	95.1	B
SMD8	PV370	85	10.1	73.9	B
SMD9	K1800	3	20.0	98.8	B
SMD10	PV370	55	7.5	47.9	E
SMD11	PV370	55	8.7	55.6	B
SMD12	Accurel PP S6/2	2	120	136.7	D
SMD13	Accurel PP V8/2 HF	1	69	119.5	B

**Table 3:** Parameters of capillary MD modules.

Module	Membrane	Number of Capillaries	Length [cm]	Area (Inside) [cm^2^]	Housing Diameter [mm]
CMD1	Accurel PP S6/2	8	65	294	12
CMD2	Accurel PP V8/2 HF	1	68	117	12

**Table 4:** Comparison of the results of the MD process obtained for different submerged MD modules.

Process	Membrane	D_in_ [mm]	Wall Thickness [µm]	T_F_ [K]	TDS [g/L]	Flux [L/m^2^h]	Ref.
VMD	Accurel PP S6/2	1.8	400	343	14.2	8.2	[15]
VMD	Accurel PP S6/2	1.8	400	343	Demi 200	9.0 6.0	[30]
VMD	Accurel PP S6/2	1.8	400	343	6.92	13.0	[55]
VMD	PTFE	0.86	415	348	1.8	4.2	[31]
VMD	PTFE	0.8	450	348	100	6.23	[32]
VMD	PVDF	0.7	250	328	demi	14.1	[13]
DCMD	Accurel PP S6/2	1.8	400	343	Demi 200	4.6 3.0	[30]
DCMD	Accurel PP S6/2	1.8	400	-	demi	4.6	[56]
DCMD	Accurel PP S6/2	1.8	400	-	-	8.2	[24]
DCMD	PVDF	0.7	250	328	demi	8.5	[13]
DCMD	PVDF	0.7	250	328	12	2.7	[57]
DCMD	PVDF	0.7	250	328	demi	7.9	[13]
